# 2-Nonanol produced by *Bacillus velezensis* EM-1: a new biocontrol agent against tobacco brown spot

**DOI:** 10.3389/fmicb.2025.1582372

**Published:** 2025-04-30

**Authors:** Xiaona Sui, Xiaobin Han, Xianbo Wang, Jun Wan, Mingxia Wen, Donglin Zhao, Yanfen Zheng, Chengsheng Zhang, Chuantao Xu, Youqiang Wang

**Affiliations:** ^1^Tobacco Research Institute of Chinese Academy of Agricultural Sciences, Qingdao, China; ^2^Zunyi Branch of Guizhou Tobacco Company, Zunyi, China; ^3^Luzhou Branch of Sichuan Tobacco Company, Luzhou, China

**Keywords:** *Alternaria alternata*, *Bacillus velezensis*, tobacco brown spot disease, volatile organic compounds, 2-nonanol, transcriptomics, carbon metabolism

## Abstract

Tobacco brown spot disease, caused by *Alternaria alternata*, poses a significant threat to crop production. Traditional control methods, particularly chemical fungicides, have raised concerns about environmental impact and resistance. Although our previous research has shown that volatile compounds produced by *Bacillus velezensis* EM-1 can effectively suppress *A. alternata*, the specific antifungal compounds and their mechanisms remain unclear. In this study, exposure to the volatiles from strain EM-1 significantly inhibited the mycelial growth and spore germination of *A. alternata*, with 2-nonanol identified as the most potent antifungal compound. Fumigation experiments revealed that 2-nonanol exhibited strong dose-dependent toxicity, with an EC_50_ of 0.1055 μL/cm^3^ and a minimum inhibitory concentration of 0.2166 μL/cm^3^. *In vivo* experiments on tobacco leaves confirmed that 2-nonanol effectively reduced tobacco brown spot disease incidence and slowed lesion expansion. Transcriptome analysis indicated that 2-nonanol downregulated the expression of genes encoding D-glucose synthesis in carbon metabolism, which limited energy acquisition by *A. alternata*. Moreover, the expression of antioxidant enzymes, including superoxide dismutase (SOD) and catalase (CAT), was markedly suppressed by 2-nonanol, thereby exacerbating cellular damage induced by oxidative stress. These findings suggest that 2-nonanol holds potential as a biocontrol agent for managing tobacco brown spot disease, underscoring the promising role of volatile organic compounds (VOCs) in the development of environmentally friendly biocontrol products.

## 1 Introduction

Tobacco brown spot, caused by *Alternaria alternata* (Fr.) Keissler, is a major fungal disease affecting tobacco. It primarily infects mature tobacco leaves, severely impacting yield and quality (Hou et al., 2016; Jing et al., 2018). Currently, chemical fungicides are the most common approach to managing tobacco brown spot disease (Xiang et al., 2022). However, due to the short harvesting interval (7–10 days), pesticide residues often accumulate on the crop. In addition, the prolonged use of chemical agents can lead to negative issues such as increased pathogen resistance and environmental pollution (Islam et al., 2024). As a result, biological control methods have garnered increasing attention in recent years, valued for their safety, efficacy, and environmental sustainability. Among biological agents, *Bacillus* species have emerged as the most widely studied and utilized biocontrol bacteria, owing to their simple nutritional needs, rapid proliferation, and production of a variety of bioactive compounds (Zhang et al., 2023). Strains such as *Bacillus siamensis* LZ88 (Xie et al., 2021), *Bacillus megaterium* strain L2 (Li et al., 2015), and *Bacillus amyloliquefaciens* HZ-12 (Xu et al., 2020) have shown promising potential for controlling tobacco brown spot.

*Bacillus* species are capable of producing a wide variety of antifungal substances, which can be categorized into ribosome-synthesized lantibiotics (Halami, 2019) or antagonistic proteins (Yi et al., 2024), non-ribosome-synthesized lipopeptide antibiotics (Platel et al., 2022), and volatile organic compounds (VOCs) (Ávila-Oviedo et al., 2024). Among them, VOCs are increasingly concerned due to their characteristics of high antifungal activity, multiple components, easy volatilization, and no residue on plant surfaces. The VOCs released by microorganisms exert various beneficial effects, including promoting plant growth and stress resistance, as well as inhibiting pathogen growth and spore germination (Awan et al., 2023; Sheikh et al., 2023).

Recent studies have revealed that *Bacillus* species can secrete various VOCs, such as aldehydes, ketones, alcohols, and phenols (Sui et al., 2022; Poulaki and Tjamos, 2023). Surovy et al. (2023) identified 39 VOCs from four *Bacillus* species, belonging to alcohols, fatty acids, ketones, aldehydes, and sulfur compounds (Surovy et al., 2023). Notably, hexanoic acid, 2-methylbutanoic acid, and phenylethyl alcohol showed inhibitory effects against *Magnaporthe oryzae* Triticum (MoT). In a separate study, Zhao et al. (2019) reported that 2,4-di-tert-butylphenol, a VOC produced by *Bacillus subtilis* CF-3, effectively prevents litchi fruit from rotting after infection by *Colletotrichum gloeosporioides* and inhibits the pathogen’s spore germination and growth (Zhao et al., 2019). Wang et al. (2022) identified 2-methylbutanoic acid and 3-methylbutanoic acid from *B. siamensis* LZ88, which exhibits strong inhibitory activity against *A. alternata* (Wang et al., 2022). Given their complex composition and diverse functions, microbial VOCs have become an area of increasing research. However, studies investigating the antifungal mechanisms of individual VOCs against *A. alternata* remain limited.

Our previous research demonstrated that the volatiles of *Bacillus velezensis* strain EM-1 exhibit strong inhibitory effects on *A. alternata* (Sui et al., 2022), but its effective components and antifungal mechanism remain unclear. Therefore, the aims of this study were identify the effective volatiles of strain EM-1 against *A. alternata*, and investigate the molecular mechanisms behind their antifungal activity. This study not only provides a novel biocontrol agent for managing tobacco brown spot but also offers valuable insights into the industrialization and commercialization of biogenic VOCs for plant disease control.

## 2 Materials and methods

### 2.1 Strains and cultivation

*B. velezensis* EM-1 (GeneBank accession number: OK090956) (Sui et al., 2022) and *A. alternata* CX06 were preserved by the National Agricultural Environmental Microbial Germplasm Resources Bank (Shandong).

PDA medium: 6 g/L of potato starch, 20 g/L of glucose, 15 g/L of agar, pH 7.0. LB medium: 10 g of tryptone, 5 g of yeast extract, 10 g of NaCl. If preparing solid medium, add 15 g of agar, pH 7.0.

### 2.2 The effect of volatiles from strain EM-1 on *A. alternata* mycelia growth

Strain EM-1 was inoculated by streaking on an LB plate. A 5 mm diameter disc of *A. alternata* was inoculated in the center of a PDA plate. The LB agar plate was placed on top of the PDA plate and sealed with PE wrap. After 3 days of constant temperature incubation at 25°C, the antifungal activity was measured. The LB agar plate without bacteria was used as the control, and the experiment was repeated three times. The antifungal rate (%) = (colony diameter of the control group – colony diameter of the treated group) / (colony diameter of the control group – 5 mm) × 100.

### 2.3 The effect of volatiles from strain EM-1 on *A. alternata* spore germination

The spore suspension of *A. alternata* was prepared as follows: A sterile plate covered with mycelium was filled with an appropriate volume of sterile water. Using a spreader, the surface of the plate was gently spread to suspend both the mycelium and spores in the sterile water. The mixture was filtered through four layers of sterile gauze, and the filtrate was collected. The spore concentration was adjusted to 2 × 10^4^ spores/mL using an inverted microscope, following the method described in previous studies (Li Q. et al., 2024). Next, 100 μL of the spore suspension was placed on a sterilized concave slide. The slide was then placed into a moisture-saturated Petri dish, with an LB agar plate covered with EM-1 cells inverted and placed on top. The control group was prepared using LB agar medium without the EM-1 cells. The petri dish was sealed with parafilm and incubated in the dark at 28°C for 6 h. After incubation, the morphological changes of the spores were observed under an inverted microscope. Ten spores were examined per field, with a total of five fields analyzed. Spore germination was defined as the presence of a germ tube at least twice the length of the spore. The inhibition rate of each treatment on spore germination and growth was calculated. Each treatment was repeated three times, and the experiment was conducted in triplicate. The spore germination inhibition rate was calculated using the following formula: Spore germination inhibition rate (%) = (number of spores germinating in the control group – number of spores germinating in the treatment group) /number of spores germinating in the control group × 100.

### 2.4 The effect of monomeric compound on *A. alternata* mycelia growth

The inhibitory effects of volatile monomer components on the mycelial growth of plant pathogenic fungi was determined using the growth rate method (You et al., 2015). Six major volatile compounds (2-Heptanone, 6-methyl-2-heptanone, 2-dodecanol, 2-decanol, 2-nonanol, and methyl-2-heptanone) produced by strain EM-1 were selected to test their effect on the growth of *A. alternata*. The volatile compounds were identified using GC–MS, with detailed information available in our previous study (Sui et al., 2022). A 20 μL volume of each pure volatile compound was directly added to the center of petri dish lid to prepare a drug plate. A 5-mm diameter disk of *A. alternata* was inoculated at the center of a PDA plate. The drug plate was then aligned with the PDA plate and sealed together with PE wrap film to prevent the escape of volatile substances. The plates were incubated at a constant temperature of 25°C for 3 days. The antifungal activity was assessed by measuring the colony growth. The drug plate, which contained no volatile substance, served as the control. Each experiment was repeated three times to ensure reliability. The antifungal inhibition rate was calculated using the following formula: The antifungal rate (%) = (colony diameter of the control group – colony diameter of the treated group) / (colony diameter of the control group – 5 mm) × 100.

### 2.5 Toxicity assay of 2-nonanol against *A. alternata*

The toxicity of 2-nonanol against *A. alternata* was also assayed by mycelial growth rate method. Different concentrations of 2-nonanol (0, 2.5, 5, 7.5, 10, 12.5 μL) were added to the center of the petri dish lid, resulting in corresponding concentrations of 0, 0.0332, 0.0663, 0.0995, 0.1327, 0.1658 μL/cm^3^. The plates were sealed with PE film and incubated upside down at 25°C for 4 days. After incubation, the growth of the mycelium was observed, and the colony diameter was measured using the cross-hatch method to calculate the antifungal rate. Each treatment was performed in triplicate. SPSS 22.0 software was used to generate a standard curve, establish a toxicity regression equation, and calculate the correlation coefficient, as well as the EC_50_ and EC_90_ values. The minimum inhibitory concentration (MIC) was defined as the lowest concentration of 2-nonanol that visibly inhibited the growth of *A. alternata*.

### 2.6 Microscopic observation of *A. alternata* mycelium

The hyphae morphology of *A. alternata* pathogen was observed using scanning electron microscopy (SEM) (Zheng et al., 2017). A 5-mm diameter disk of *A. alternata* was placed at the center of PDA medium and exposed to 0.2110 μL/cm^3^ l (2 × EC_50_) of 2-nonanol, and then incubated in the dark at 25°C for 4 days. After incubation, the collected hyphae were fixed in 2.5% glutaraldehyde solution at 4°C for 4 h. The fixed samples were then washed six times with 0.01 M phosphate buffer solution. Gradient dehydration was performed by sequentially immersing the samples in ethanol solutions with increasing concentrations: 30, 50, 70, 80, 90, 95, and 100%, with each step lasting 30 min. Finally, the samples were transferred to isoamyl acetate, subjected to critical point drying using carbon dioxide, and then coated with gold. The morphology of the hyphae was observed using a scanning electron microscope.

### 2.7 Inhibitory effect of 2-nonanol against *A. alternata* on isolated leaves

The *in vivo* leaf bioassay for assessing the efficacy of 2-nonanol against tobacco brown spot disease was performed using spore suspension hanging drop inoculation. The strain EM-1 was inoculated densely on an LB agar plate, with a blank LB agar plate serving as the control. Two appropriately sized mature tobacco leaves (variety NC89) were placed at the bottom of a petri dish, with a wet cotton ball positioned around the petiole to maintain moisture. After spraying the leaf surface with sterile water, several inoculation points were selected along both sides of the main vein. Wounds were made using needles, and 100 μL of the brown spot pathogen spore suspension (1 × 10^6^ spores/mL) was applied to each wound using the hanging drop method. Each treatment was performed in 10 petri dishes, with three replicates per treatment. A total concentration of 0.211 μL/cm^3^ (2 × EC_50_) of 2-nonanol was added to the edge of the petri dish, which was then sealed with parafilm. The plates were incubated at 28°C in the dark for 80 h. After the incubation period, disease spot development on the leaves was assessed, and the inhibition rate was calculated using the following formula: Disease spot spread inhibition rate (%) = (diameter of disease spots in the control group – diameter of disease spots in the treatment group) /diameter of disease spots in the control group × 100.

### 2.8 RNA sequencing analysis

The 2-nonanol treatment was performed as described above to achieve a final concentration of 0.1055 μL/cm^3^ (EC_50_). A 5-mm disk of *A. alternata* pathogen was placed at the center of a PDA plate, with one disk per plate. The plates were then incubated in the dark at 25°C for 3 days. Following the incubation period, the mycelia were collected, wrapped in aluminum foil, and immediately flash-frozen in liquid nitrogen. Control samples consisted of mycelium grown on blank PDA plates. Each treatment was replicated three times.

Total RNA was extracted from the samples using TRIzol® Reagent according to the manufacturer’s protocol. RNA sequencing libraries were constructed using the TruSeq™ Stranded Total RNA Library Prep Kit and TruSeq PE Cluster Kits, and sequencing was performed on the Illumina NovaSeq 6,000 platform. Library construction and high-throughput sequencing were outsourced to Majorbio Co., Ltd., Shanghai, China. Raw RNA-seq data were preprocessed using Trimmomatic (v.0.39) (Bolger et al., 2014) to filter low-quality reads and adapter sequences. To identify differentially expressed genes (DEGs) between the control and treated samples, the high-quality reads were aligned to the *A. alternata* (GCA_001572055.1) genome using HISAT2 (v.2.2.1) (Kim et al., 2019) with default parameters. Transcript expression levels were quantified using RSEM (v.1.3.3) (Li and Dewey, 2011), with the resulting expression values provided as fragments per kilobase of transcript per million mapped reads (FPKM). The Log_2_ fold change (LFC) and false discovery rate (FDR) of DEGs between control and treated samples were calculated using DESeq2 (v.1.24.0) (Love et al., 2014). DEGs were considered significant if |LFC| ≥ 1 and *p*-value < 0.05. DEGs annotation information was obtained by performing a blast search against the gene ontology (GO) and Kyoto Encyclopedia of Genes and Genomes (KEGG) database pipelines. GO and KEGG pathway enrichment analyses of the DEGs were performed using the Goatools (v.0.6.5) (Klopfenstein et al., 2018) and Python scipy (v.1.13.0) (Virtanen et al., 2020) software, respectively (*P*_FDR < 0.05).

### 2.9 RT-qPCR

To verify the accuracy and reliability of the transcriptome sequencing results, ten genes were selected for Reverse Transcription Quantitative PCR (RT-qPCR) validation. These genes included *AALT_g4954, AALT_g2578, AALT_g11095, AALT_g10361, AALT_g9032, AALT_g10686, AALT_g4845, AALT_g6409, AALT_g7589, and AALT_g11896*, with *β-Tubulin* used as the internal reference gene. The sample RNA was reverse transcribed into cDNA using the HiScript II 1st Strand cDNA Synthesis Kit (Vazyme Biotech, China). The resulting cDNA was then used as the template for RT-qPCR. The RT-qPCR was performed with SYBR Premix Ex TaqTM (TaKaRa) using the 7,500 Real-Time PCR System (Applied Biosystems, USA). The 20 μL reaction mixture contained 10 μL of 2 × SYBR Green Premix Ex Taq, 0.4 μL of each forward and reverse primer, 0.4 μL of ROX Reference Dye II, 2 μL of cDNA template, and distilled water. Primer sequences are listed in Table 1, with all primer synthesized by Sangon Biotech (Shanghai) Co., Ltd. The relative expression levels of the target genes were calculated using the 2^-ΔΔCt^ method (Livak and Schmittgen, 2001).

**Table 1:** Primers used for RT-qPCR analysis in this study.

Gene name	Forward primers	Reverse primers
*AALT_g4954*	AAAGGAGTATGAGTTTTGGGTGAA	GAGTCTCGTTGAGTGGCTGTGT
*AALT_g2578*	AACGGCGAGGTTCTATTTGA	TAGAGGTTGGGGTTGTCAGG
*AALT_g11095*	GCGTTACTGGGACGAGGTTG	GAATGACAGGTCGGTTTGGG
*AALT_g10361*	GGTCACATCAACCACTCGCTC	GAACTTGTCCTCATCACCCCA
*AALT_g9032*	CCACGGCACCTTTGTTTCT	GGCAACAGTGGAGAATCGC
*AALT_g10686*	CACTGGCAACAACAGCATTCC	GCCGTTGGGTGACAGGGA
*AALT_g4845*	ACCGTGTCTGGAACCCCTT	ATGTTCCTGAGGCGGCAC
*AALT_g6409*	CGAATACGCCACCCTCACA	CAACACCGCAGCCAACATC
*AALT_g7589*	GTCCCGCCCACCACACA	CCTTCGCCCGCATAACCT
*AALT_g11896*	TACAATCGTGTCAACGGCAGT	TGCGTAGCCTCCCAGTCAG
*β-Tubulin*	TTCAACGAAGCCTCCAACAAC	GTGCCGGGCTCGAGA

### 2.10 Statistical analysis

The data presented in the figures are expressed as the mean ± standard deviation (SD). Data analysis and visualization were performed using SPSS 16.0 software (IBM, USA) and Prism 8.40 (GraphPad, USA). To assess statistical significance, one-way analysis of variance (ANOVA) and unpaired Student’s *t*-test were used. Significance levels were defined as *p < 0.05, **p < 0.01, and ***p < 0.001.

## 3 Results

### 3.1 Effects of volatiles from *B. velezensis* EM-1 on mycelial growth and spore germination of *A. alternata*

**Figure 1:**
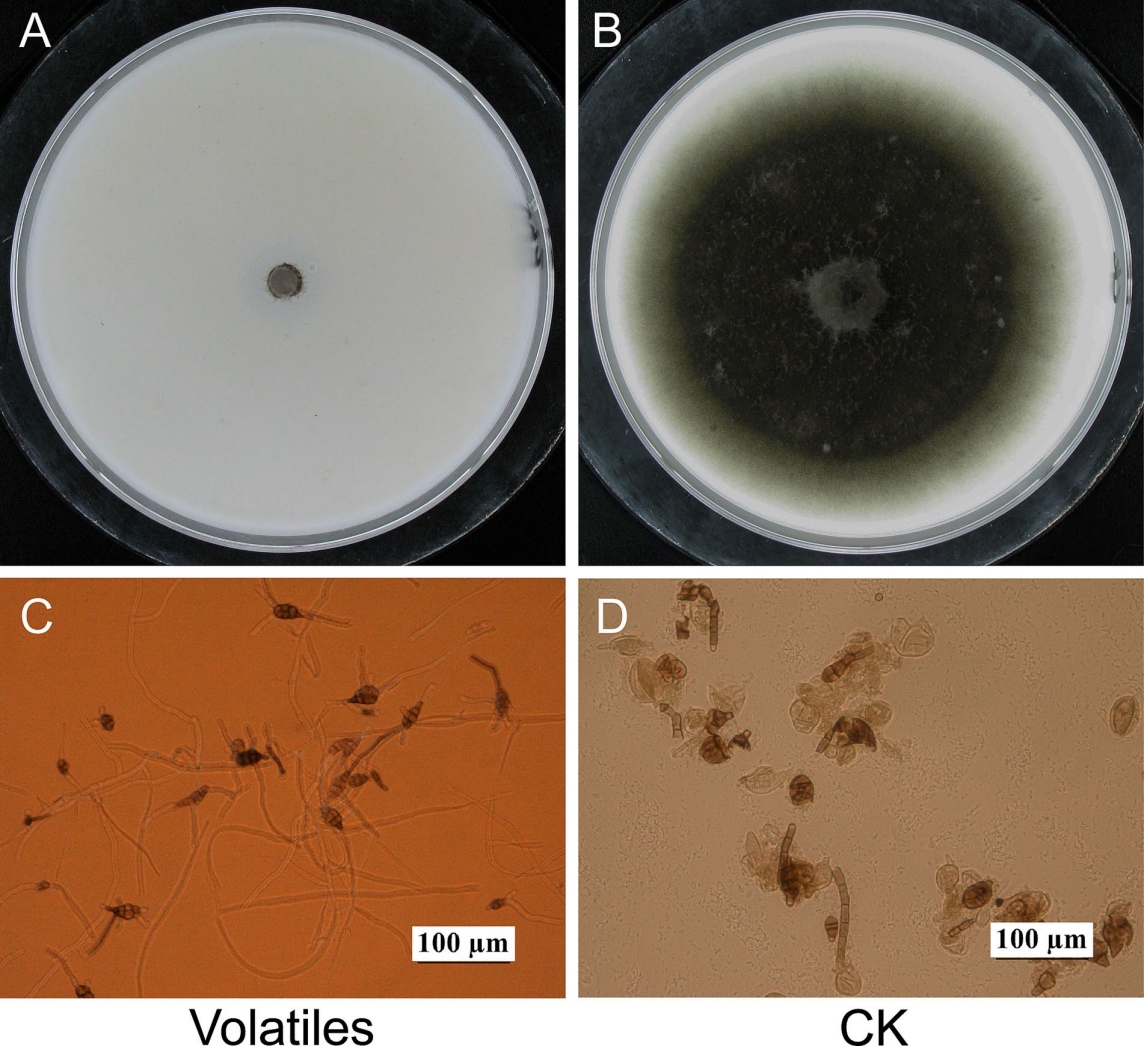
Effects of volatiles from *Bacillus velezensis* EM-1 on *A. alternata.* Mycelial growth of *A. alternata* treated **(A)** and untreated **(B)** with volatiles. Spores germination of *A. alternata* treated **(C)** and untreated **(D)** with volatiles.

The volatiles of strain EM-1 strongly inhibited the growth of the pathogen *A. alternata* (Figure 1). Compared to the control treatment (CK), the colonies of *A. alternata* exposed to the volatiles showed virtually no growth, with an inhibition rate approaching 100% (Figures 1A,B). Microscopic observation showed that the conidial germination of *A. alternata* treated with the volatiles was completely inhibited, and the germ tubes that did germinate grew slowly, were short and deformed, with most conidial structures damaged (Figure 1C). However, the conidia and mycelial development of *A. alternata* without volatile treatment were normal (Figure 1D). These results indicated that the volatiles produced by strain EM-1 had a strong inhibitory effects on *A. alternata*.

### 3.2 Inhibitory effects of different volatile components on the mycelia growth of *A. alternata*

**Figure 2:**
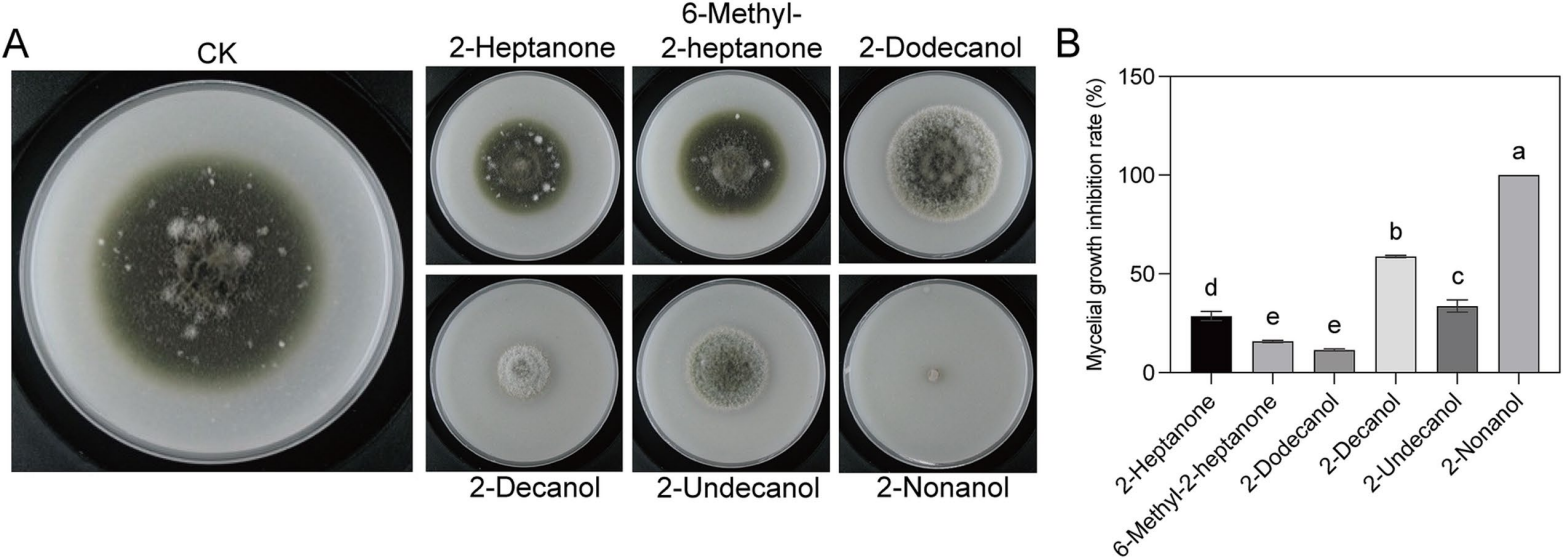
Inhibitory effects of six volatile compounds on *A. alternata*. **(A)** Colony morphologies with different volatiles treatment. **(B)** Inhibition rate of six volatile compounds on *A. alternata.* Different letters represent significant differences (*p* < 0.05) using one-way analysis of variance (ANOVA) with Tukey’s test.

In our previous study, six volatile compounds, including 2-heptanone, 6-methyl-2-heptanone, 2-dodecanol, 2-decanol, 2-nonanol, and methyl-2-heptanone, produced by the strain EM-1 were obtained using GC–MS (Sui et al., 2022). To investigate which volatile compound exerts the effect, we tested the inhibitory activity of six volatile compounds against the tobacco brown spot pathogen (Figure 2). The results indicated that all six volatiles exhibited inhibitory effects on the growth of the pathogen *A. alternata* (Figure 2A), with mycelial growth inhibition rates ranging from 11.46 to 100% (Figure 2B). Among the tested compounds, 2-nonanol exhibited the strongest antifungal activity, with an inhibition rate of 100%, followed by 2-decanol (58.59%). However, the inhibition rates of the other four substances were all below 30%. In addition to significantly inhibiting mycelial growth, the volatiles from strain EM-1 also affected colony morphology (Figure 2A). Under CK treatment, the mycelium of *A. alternata* was dense, grew vigorously, and the colony appeared black. In contrast, the mycelium treated with 2-nonanol and 2-decanol was more sparse, grew slowly, and likely inhibited melanin formation, resulting in grayish-white colonies. This suggests that 2-nonanol was the key active compounds in the volatiles to inhibit the pathogen *A. alternata*.

### 3.3 Toxicity of 2-nonanol against *A. alternata*

**Figure 3:**
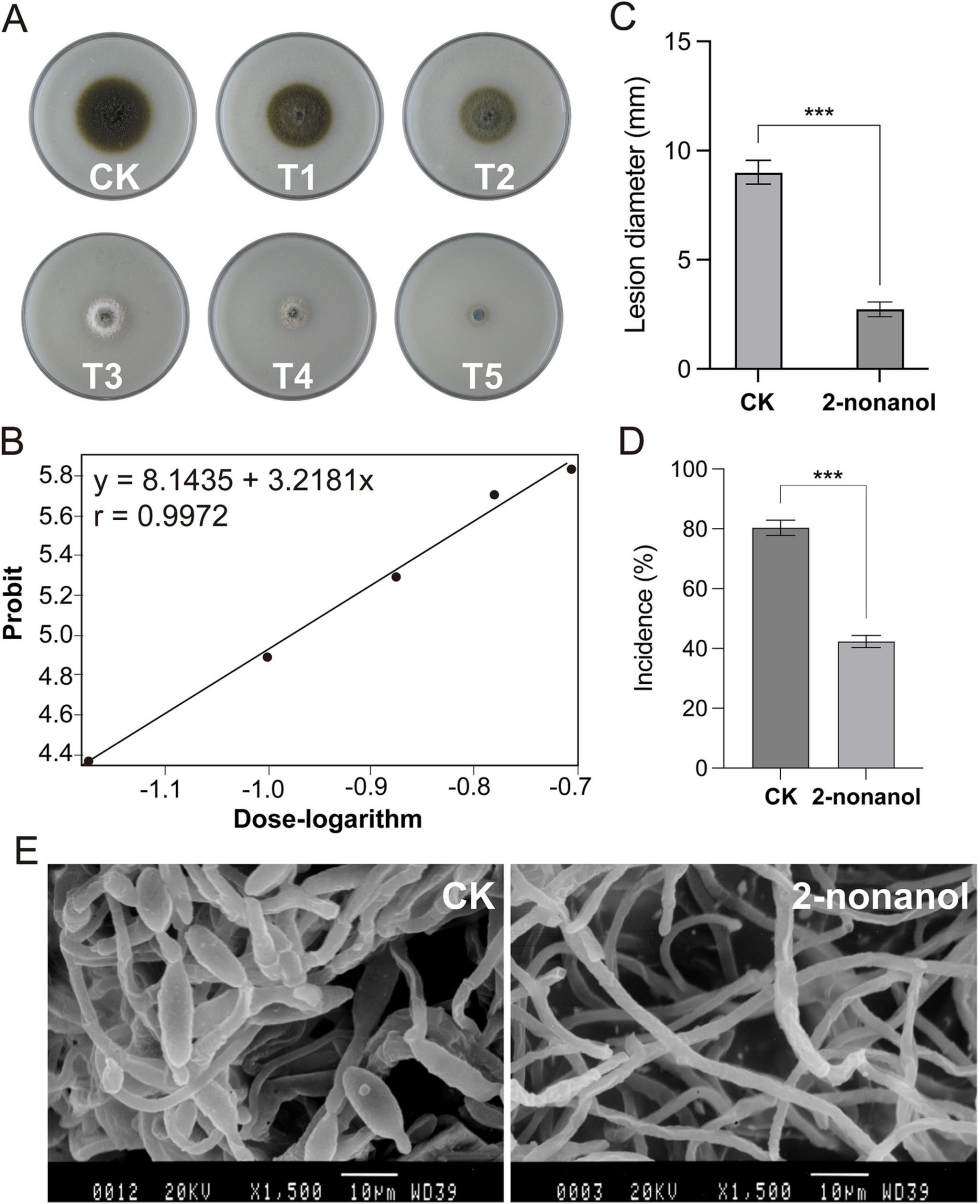
Toxicity of 2-nonanol against *A. alternata.* **(A)** Inhibitory effects of varying doses of 2-nonanol on *A. alternata*. 2-nonanol dose (uL/cm^3^): 0 (CK), 0.0332(T1), 0.0663 (T2), 0.0995 (T3), 0.1327 (T4), 0.1658 (T5). **(B)** Toxicity regression curve of 2-nonanol. **(C)** Lesion diameter of brown spot *in vivo*. **(D)** The incidence rate of brown spot disease. **(E)** Microstructure of the mycelia and spores of *A. alternata*. Asterisks indicate significant differences determined by two-sided Student’s *t*-test (***p < 0.001).

Fumigation experiments were conducted on *A. alternata* by using 2-nonanol with different supply concentrations. The results showed that as the dosage of 2-nonanol increased, the inhibitory effects on *A. alternata* became more significant (Figure 3A). Based on the colony diameter, the half-maximal effective concentration (EC_50_) of 2-nonanol against tobacco brown spot disease was calculated to be 0.1055 μL/cm^3^ (95% confidence interval: 0.1055 ~ 0.1088 μL/cm^3^). The absolute value of the toxicity correlation coefficient for 2-nonanol against tobacco brown spot disease was 0.9972, which was greater than 0.90, indicating a strong linear correlation between the variables in the toxicity regression equation. 2-Nonanol positively correlates with the inhibition of brown spot disease colony diameter. As the effective concentration increases, the inhibition rate increases, and the inhibitory effect becomes stronger. The toxicity regression equation for 2-nonanol against tobacco brown spot disease was y = 3.2182x + 8.1435. The minimum inhibitory concentration (MIC) of 2-nonanol against tobacco brown spot disease was 0.2166 μL/cm^3^ (Figure 3B).

### 3.4 *In vivo* control effect of 2-nonanol on tobacco brown spot disease

To validate the efficacy of 2-nonanol against tobacco brown spot disease, we conducted an inoculation experiment of the pathogen *A. alternata* on tobacco leaves with and without 2-nonanol. The results showed that exogenous application of 2-nonanol significantly inhibited the spread of tobacco brown spot disease lesions (Figures 3C,D). In the untreated leaves, the disease incidence was 88.33%, and the lesions expanded into near-circular brown necrotic spots with an average diameter of 8.32 mm. In comparison, after exposure to the 2-nonanol treatment, the incidence dropped to 60.80%, with slower lesion expansion, measuring an average of 2.85 mm. This resulted in a 69.37% reduction in lesion expansion. SEM analysis of the ultrastructure of *A. alternata* hyphae further confirmed the inhibitory effects of 2-nonanol (Figure 3E). The hyphae in the CK treatment appeared normal, with uniform thickness and healthy spore production. However, following 2-nonanol treatment, the hyphae exhibited significant abnormalities, including uneven thickness, irregular twisting, and no conidial formation. In addition, a large number of spindle-shaped conidia were visible in the CK treatment, but no conidia were observed in the 2-nonanol treatment. These results suggest that exogenous application of 2-nonanol effectively controlled *A. alternata*-induced tobacco brown spot disease.

### 3.5 Transcriptional response of *A. alternata* to 2-nonanol

**Figure 4:**
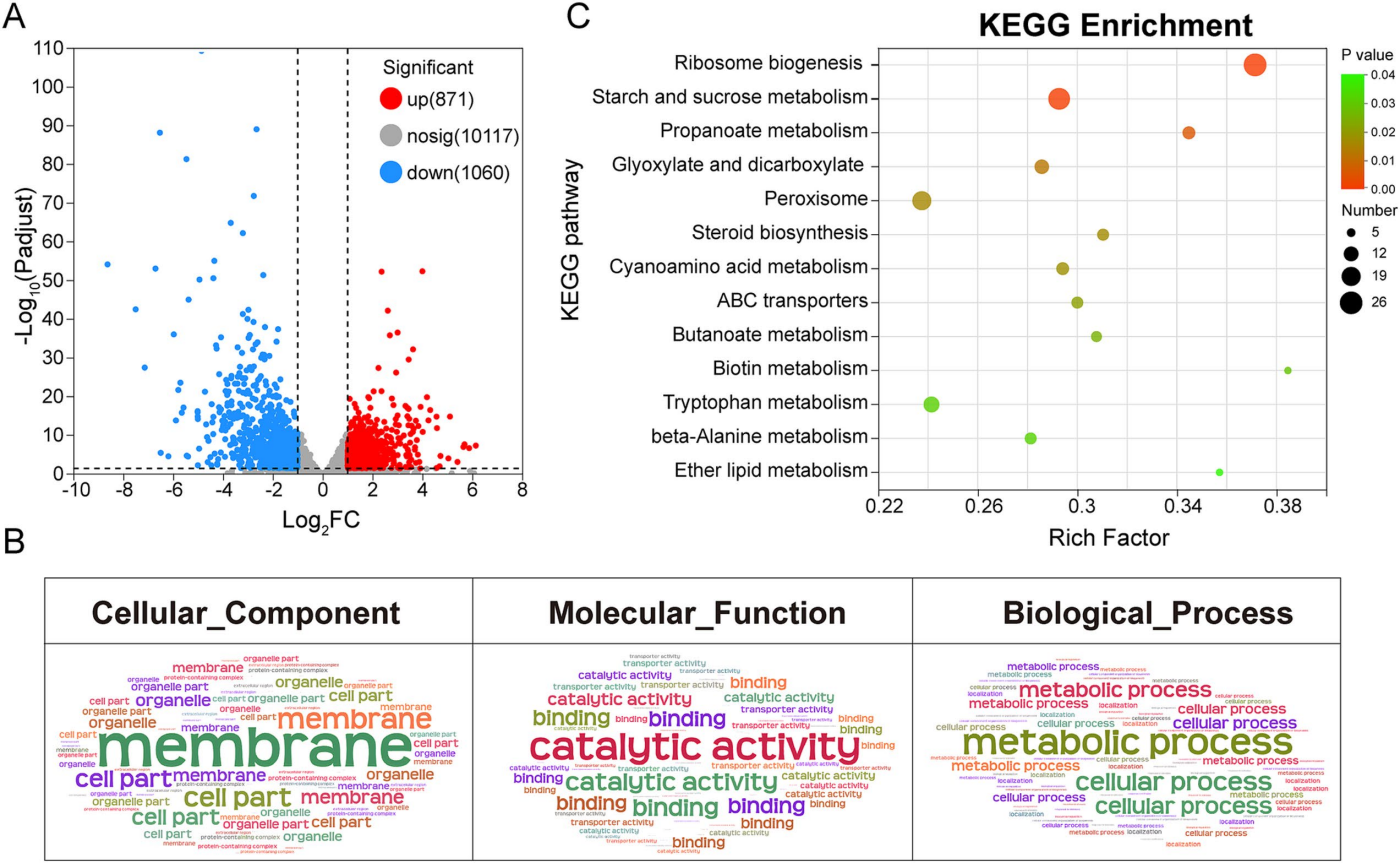
Transcriptional responses of *A. alternata* to 2-nonanol. **(A)** Volcano plot of differentially expressed genes. **(B)** GO annotation analysis. **(C)** KEGG enrichment analysis.

To further elucidate the molecular mechanism underlying the antimicrobial activity of 2-nonanol, we performed RNA-seq analysis on *A. alternata* with or without 2-nonanol (Figure 4). The results revealed that the addition of 2-nonanol identified a total of 1,931 differentially expressed genes (DEGs), including 871 upregulated and 1,060 downregulated genes (Figure 4A). GO annotation analysis of the DEGs through cellular processes, molecular functions, and biological processes showed that membrane, catalytic activity, and metabolic processes were the core terms responsive to 2-nonanol (Figure 4B). Furthermore, KEGG enrichment analysis indicated that the DEGs were primarily involved in pathways such as ribosome biogenesis, starch and sucrose metabolism, and peroxisome metabolism. Among them, the largest number of genes is enriched in ribosome biosynthesis and starch and sucrose metabolism (Figure 4C). These findings suggest that 2-nonanol modulates the transcriptional activity of *A. alternata*.

### 3.6 2-Nonanol reconfigures gene expression in *A. alternata*

**Figure 5:**
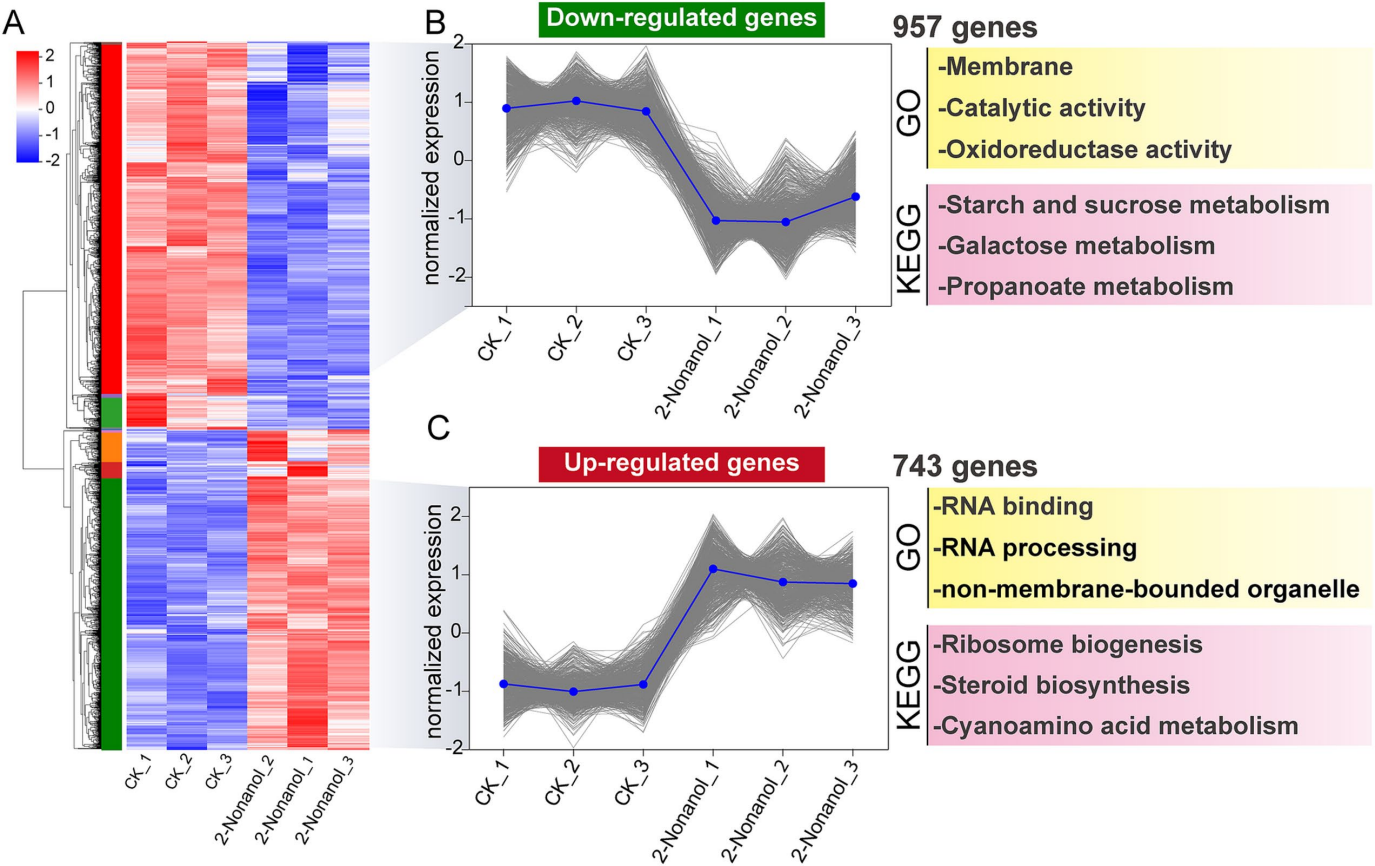
Hierarchical clustering analysis of differentially expressed genes. **(A)** Hierarchical clustering analysis. **(B,C)** Subclusters of downregulated and upregulated genes.

Hierarchical clustering analysis was conducted on DEGs exhibiting similar expression patterns, identifying key subclusters of 743 upregulated and 957 downregulated genes (Figure 5A). GO and KEGG enrichment analyses revealed that the upregulated genes were associated with biological processes such as ribosome biogenesis, RNA processing, steroid biosynthesis, and cyanoamino acid metabolism (Figure 5C). However, genes involved in C metabolism and oxidoreductase metabolism were significantly downregulated in response to 2-nonanol (Figure 5B). These findings suggest that 2-nonanol exerts its influence on *A. alternata* by modulating a variety of physiological and metabolic pathways.

**Figure 6:**
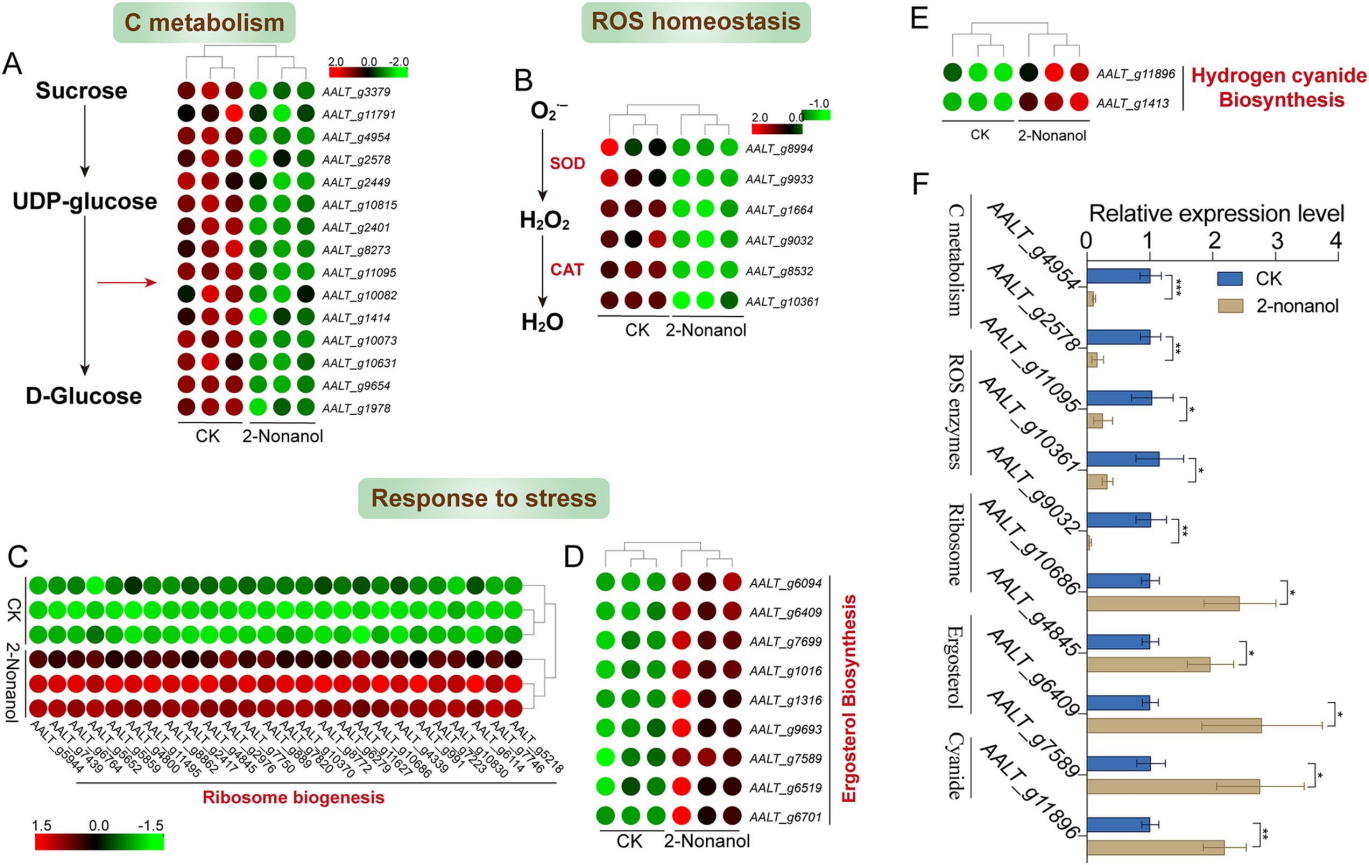
Gene expression changes in response to 2-nonanol. **(A)** C metabolism. **(B)** ROS-scavenging enzymes. **(C)** Ribosome biogenesis. **(D)** Ergosterol. **(E)** Cyanide-related compounds. **(F)** RT-qPCR analysis of the genes expression levels. Asterisks indicate significant differences determined by two-sided Student’s *t*-test (*p < 0.05, **p < 0.01, and ***p < 0.001).

We focused on the changes in expression levels of key genes responding to 2-nonanol (Figure 6). The results revealed that 2-nonanol significantly suppressed the expression of genes involved in D-glucose biosynthesis, thereby disrupting C metabolism processes (Figure 6A). Additionally, the expression of genes encoding superoxide dismutase (SOD) and catalase (CAT) enzymes was markedly reduced, leading to excessive accumulation of reactive oxygen species (ROS) within the pathogen (Figure 6B). In contrast, genes associated with ribosome biogenesis were strongly upregulated, suggesting that *A. alternata* synthesizes substantial amounts of stress-response proteins (e.g., antioxidant enzymes, membrane repair proteins) to mitigate the stress induced by 2-nonanol (Figure 6C). Furthermore, the upregulation of genes encoding ergosterol (*ERGs*), an essential component of fungal cell membranes, indicates potential damage to the pathogen’s cell membrane (Figure 6D). Lastly, 2-nonanol treatment was observed to induce the accumulation of intracellular toxic substances, such as cyanide-related compounds, which likely contribute to the death of *A. alternata* (Figure 6E). RT-qPCR analysis revealed that 2-nonanol significantly decreased the expression levels of genes associated with C metabolism and ROS scavenging enzymes, while genes related to stress responses were upregulated (Figure 6F). This further supports the reliability of the RNA-seq results.

## 4 Discussion

This study demonstrates that the volatile compounds produced by *B. velezensis* EM-1 exhibit a strong inhibitory effects on pathogen *A. alternata*. Through physiological and *in vivo* assays, we identified 2-nonanol as the key active component responsible for this inhibition and explored its antifungal mechanism via transcriptomic analysis. This research provides a crucial theoretical foundation for the green management of tobacco brown spot disease.

Since the report by Fiddaman and Rossall (1994) on the secretion of antifungal volatile compounds by *Bacillus subtilis*, extensive research has been conducted on the antifungal activity of volatiles produced by *Bacillus* genus (Fiddaman and Rossall, 1994). For instance, the volatiles produced by *Bacillus tequilensis* XK29 have been shown to inhibit mycelial development, conidial formation, and metabolic activity in *Botrytis cinerea* (Guo et al., 2023). Similarly, the VOCs released by *Bacillus velezensis* LT1 significantly impede mycelial growth and sclerotial germination of *Sclerotium rolfsii*, while also disrupting the morphological integrity of fungal hyphae (Tang et al., 2024). In this study, the volatiles from *B. velezensis* EM-1 exhibited varying degrees of antifungal and teratogenic effects on the mycelial and colony growth, as well as spore germination, of *A. alternata* (Figures 1, 2). These results suggest that the production of extracellular antifungal volatiles by strain EM-1 was closely linked to its antagonistic activity. VOCs are excellent informational chemicals, with their ability to diffuse easily allowing their activity to range from localized to more distant interactions (Wheatley, 2002). In practical applications, such as tobacco cultivation, these strains can be sprayed directly onto leaves, allowing the antifungal volatiles to disperse in the surrounding environment and provide effective protection against pathogen infections.

To date, numerous antifungal substances have been identified from the volatiles produced by *Bacillus* species. Hassan et al. reported that the volatile compound 3-methyl-1-butanol, derived from *Bacillus licheniformis* 350–2, significantly inhibited the growth, sporulation, and mycotoxin accumulation of *Aspergillus* spp. and *Penicillium* spp. (Ul Hassan et al., 2019). Likewise, the volatiles 2-methylbutanoic acid and 3-methylbutanoic acid, produced by *Bacillus siamensis* LZ88, demonstrated strong inhibitory activity against the mycelial growth of *A. alternata*, with IC_50_ values of 83.10 mg/mL and 104.19 mg/mL, respectively (Wang et al., 2022). Here, we identified 2-nonanol as the key antifungal component in the volatiles from *B. velezensis* EM-1, which effectively inhibited the mycelial growth of *A. alternata* with an EC_50_ of 0.1055 μL/cm^3^ (Figures 2, 3B). This compound was notably more potent than 2-methylbutanoic acid and 3-methylbutanoic acid in suppressing mycelial growth. Futhermore, 2-nonanol inhibited spore production, disrupted the mycelial structure, and suppressed lesion expansion of *A. alternata* on isolated leaves (Figures 3C–E). Interestingly, while the volatile 2-heptanone produced by *B. velezensis* EM-1 was shown to inhibit *Ralstonia solanacearum* in previous studies (Sui et al., 2022), it did not exhibit antifungal activity against *A. alternata* in this work. This finding highlights the specificity of antifungal activity among *Bacillus* VOCs toward different fungal pathogens. Similarly, 3-methyl-1-butanol, which inhibits the growth of *Botrytis cinerea* mycelia and conidial germination, showed no antifungal effect against *A. alternata* (Marzouk et al., 2021; Zou et al., 2022).

The microbial volatile 2-nonanol has been reported to exhibit inhibitory effects against various plant pathogens (Boukaew et al., 2024; Song et al., 2024). However, research on its antifungal mechanism remains limited, and no systematic studies have elucidated its inhibitory effects on *A. alternata*, the pathogen responsible for tobacco brown spot disease. Our transcriptomic analysis revealed that 2-nonanol downregulated gene expression associated with the starch and sucrose metabolism and peroxisome pathways in *A. alternata* (Figures 4, 5B). Starch and sucrose metabolism constitutes the core of carbohydrate metabolism and serves as a crucial pathway for energy acquisition in fungi. Previous studies have demonstrated that disrupting carbohydrate metabolism impairs the respiratory chain and energy supply, thereby inhibiting fungal growth, as observed in *Cryptococcus neoformans* (Orrapin et al., 2021) and *Fusarium oxysporum* (Cao et al., 2022). Specifically, 2-nonanol suppressed the expression of key enzyme genes involved in substrate utilization (*α*-glucosidase, glycoside hydrolase), glycogen synthesis (glycogen synthase), and glycoside biosynthesis (glycosyl transferase) in the starch and sucrose metabolism pathway, thereby disrupting energy metabolism and restricting fungal growth (Figure 6A). Oxidative damage caused by excessive ROS production is a common antifungal mechanism of fungicides and plays a crucial role in the toxicity of 2-nonanol (Song et al., 2024). In response to 2-nonanol treatment, the expression of genes encoding catalase and superoxide dismutase in *A. alternata* was downregulated, impairing peroxisomal ROS scavenging capacity and exacerbating oxidative stress (Figure 6B). This finding aligns with reports by Boukaew et al. (2024), which showed that 2-nonanol suppresses antioxidant enzyme activity in *Penicillium digitatum*. Moreover, cyanide is a potent inhibitor of the respiratory chain, and 2-nonanol inhibits the mitochondrial respiratory chain of *A. alternata* by upregulating cyanide synthesis (Figure 6E), resulting in electron leakage within the electron transport chain and the excessive accumulation of ROS (O₂^−^) (Black et al., 2021). These results suggest that the inhibitory effect of 2-nonanol on *A. alternata* may be driven by disruptions in carbohydrate metabolism and redox system imbalance.

Research has indicated that the ribosome may serve as a potential target for antifungal activity (Bhabhra et al., 2008). For instance, baicalein induces apoptosis in *Candida auris* by inhibiting ribosome synthesis pathways (Li C. et al., 2024). However, we found that 2-nonanol upregulated the expression of genes related to ribosome synthesis in *A. alternata*, which are primarily involved in the processing and assembly of the 40S and 60S ribosomal subunits (Figure 6C). This suggests that the ribosome pathway is not the target of 2-nonanol’s antimicrobial effect, but rather a biological process responding to drug stimulation. In addition, the upregulation of ribosome biosynthesis increases the demand for C-skeletons and energy (Figure 6A), further disrupting cellular metabolic homeostasis. Many antifungal agents, such as azoles, act by inhibiting key enzymes in the ergosterol biosynthesis pathway (e.g., *ERG11* and *ERG3*, which catalyze the conversion of lanosterol to ergosterol) (Doorley et al., 2023; Hartuis et al., 2024). As observed in previous studies (Hunsaker et al., 2021; Choudhary et al., 2023), in this study, 2-nonanol attempts to circumvent the drug’s action by upregulating the expression of the *ERG* gene family (including *ERG2, ERG3, ERG4, ERG6, ERG25*), thereby maintaining ergosterol synthesis. Ergosterol is a critical component of fungal membranes, playing a vital role in regulating membrane fluidity and controlling the cell cycle (Liu et al., 2019; Perczyk et al., 2020). Furthermore, RT-qPCR validation confirmed the reliability of the transcriptome results, with the gene expression response to 2-nonanol aligning with the RNA-seq findings (Figure 6F). In short, *A. alternata* was sensitive to the volatile compound 2-nonanol, which exerted its antifungal effects by regulating fungal C metabolism and ROS homeostasis.

## 5 Conclusion

In summary, this study demonstrated that the volatiles from *B. velezensis* EM-1, especially 2-nonanol, effectively inhibited the mycelial growth and spore germination of *A. alternata*, the pathogen responsible for tobacco brown spot disease. Exposure to strain EM-1 volatiles led to nearly complete inhibition of mycelial growth and significant morphological damage to the pathogen. Among the tested compounds, 2-nonanol showed the strongest antifungal activity, with an EC_50_ of 0.1055 μL/cm^3^ and a MIC of 0.2166 μL/cm^3^. *In vivo* experiments further confirmed that 2-nonanol significantly reduced disease incidence and lesion expansion on tobacco leaves. Transcriptomic analysis revealed that 2-nonanol induced significant changes in gene expression, particularly in pathways related to starch and sucrose metabolism, oxidative stress response, and ribosome biogenesis. Notably, 2-nonanol disrupted *A. alternata’s* C metabolism and antioxidant defense mechanisms, leading to cellular damage and pathogen death. These findings highlight the potential of 2-nonanol as a promising biocontrol agent for managing tobacco brown spot disease. Further investigation is needed to evaluate its field efficacy and its application in integrated pest management strategies. The molecular insights gained from this study provide a deeper understanding of the mode of action of 2-nonanol and its role in modulating key metabolic pathways in fungal pathogens.

## Data Availability

The original contributions presented in the study are included in the article/supplementary material, further inquiries can be directed to the corresponding authors.
